# Climate Change Likely to Facilitate the Invasion of the Non-Native Hydroid, *Cordylophora caspia*, in the San Francisco Estuary

**DOI:** 10.1371/journal.pone.0046373

**Published:** 2012-10-11

**Authors:** Mariah H. Meek, Alpa P. Wintzer, William C. Wetzel, Bernie May

**Affiliations:** 1 Deptartment of Animal Science, University of California-Davis, Davis, California, United States of America; 2 Center for Watershed Sciences, University of California-Davis, Davis, California, United States of America; 3 Center for Population Biology, Deptartment of Evolution and Ecology, University of California-Davis, Davis, California, United States of America; University of Western Australia, Australia

## Abstract

Climate change and invasive species can both have negative impacts on native species diversity. Additionally, climate change has the potential to favor invasive species over natives, dealing a double blow to native biodiversity. It is, therefore, vital to determine how changing climate conditions are directly linked to demographic rates and population growth of non-native species so we can quantitatively evaluate how invasive populations may be affected by changing conditions and, in turn, impact native species. *Cordylophora caspia*, a hydrozoan from the Ponto-Caspian region, has become established in the brackish water habitats of the San Francisco Estuary (SFE). We conducted laboratory experiments to study how temperature and salinity affect *C. caspia* population growth rates, in order to predict possible responses to climate change. *C. Caspia* population growth increased nonlinearly with temperature and leveled off at a maximum growth rate near the annual maximum temperature predicted under a conservative climate change scenario. Increasing salinity, however, did not influence growth rates. Our results indicate that *C. caspia* populations in the SFE will benefit from predicted regional warming trends and be little affected by changes in salinity. The population of *C. caspia* in the SFE has the potential to thrive under future climate conditions and may subsequently increase its negative impact on the food web.

## Introduction

Climate change and biological invasions have both reduced native species diversity [1], [2]. Additionally, climate change has the potential to favor invasive species over natives, dealing a double blow to native species [3]. It is important to understand interactions between invasive species and climate change to better predict how the two forces work together to disrupt native communities. It is, thus, vital to determine how changing climate is directly linked to demographic rates and population growth of non-native species so we can quantitatively evaluate how a species may be affected [3], [4]. Climate factors can play an important role in invasions if they work to increase reproductive output. For example, increases in temperature can increase rates of dispersal and development, leading to faster spread and higher abundances [5]. Additionally, climate change can create opportunities for non-natives to move into areas that were previously uninhabitable [5], [6].

One major obstacle to predicting the future abundance of invasive species under climate change is determining the relationship between growth rates and the environmental variables predicted to change [7], [8]. Schwenk et al. [9] noted the need for work on the basic understanding of the link between organisms, environmental conditions, and their population growth parameters as one of the top five challenges in organismal biology. The influence of climate change on a species' population size will depend on the shape of the relationship between population growth rate and climate variables over the range of present and future values for those variables. Over the full theoretical range of any important environmental variable, such as temperature, population growth rates will reach a maximum at optimal environmental values and drop to zero at extreme low and high values. In order to predict the effects of climate change on populations, it is necessary to determine where on the growth curve the population currently is and how its position may shift with change in variables such as temperature. What the shape of the relationship is, where a population currently is on the curve, and how this position will shift under climate change, will play a large role in determining if the population will persist in the future, increase in abundance, or decrease and go locally extinct. Researchers can use these relationships to predict how species' abundances and distributions may change with climate change [10].

In this study, we experimentally determined the shape of the relationship between population growth rate and climate variables predicted to change for *Cordylophora caspia* (Pallas 1771), an important, yet understudied non-native hydroid found worldwide [11], [12]. *C. caspia* is presumed native to the Ponto-Caspia region (Pallas 1771), but has invaded freshwater and brackish habitats globally, including the Baltic Sea [13], throughout Europe [14]–[17],the east coast of the United States [18], the Great Lakes [19], and the west coast of the United States, including the Puget Sound and the San Francisco Bay [11], [12], [20], [21]. This distribution is particularly expanding within the United States [12]. In systems it invades, *C. caspia* can have serious negative economic impacts [22], [23] including biofouling of power plant pipes [12], [24] and drinking water treatment plants [22]. Additionally, it is a voracious predator, feeding largely on zooplankton, and has the potential to negatively impact the food webs it invades [12], [18], [25].

Hydroids may drastically alter food web processes if they become abundant in areas where they are not native members of the trophic community. They can play strong roles as predators, with some estimates placing their ingestion rates among the highest of suspension feeding organisms [26]. The importance of hydroids in the transfer of energy from pelagic to benthic systems is often overlooked due to their relatively small size and low abundance; however hydroid species composing less than 0.5% of the benthic community biomass have been estimated to capture approximately 10% of the annual algal production [27]. Colonies of *Eudendrium racemosum*, another colonial hydroid, must consume prey equivalent to 7.2% of their own biomass daily to meet their metabolic requirements [28].

*C. caspia* is an abundant non-native species in the SFE and is found in brackish water habitats, such as Suisun Marsh. It has density estimates in some areas of Suisun Marsh up to 626,000 hydranths/m^2^ (Wintzer, AP, unpublished data). Suisun Marsh provides valuable rearing and spawning habitat for many important planktivorous fishes [29], [30]. Populations of some of these fishes have been experiencing drastic declines in recent years [31]. It is thought that competition with non-natives may be one of the causes for the observed declines [31]–[33].

Despite its potential importance, relatively little is known about the ecology of *C. caspia*. Even less is known about how the abundance and distribution of *C. caspia* will change under future climate change. Climate change scenarios predict an increase of 2–6 C in mean annual air temperature across California [34], [35], an increase in variability in salinity regimes in the SFE [36], and more frequent extreme climatic events [37]. The scenarios for the region predict varied outcomes, with some native species likely to decline due to raising temperatures and others to benefit from increased variability in hydrologic regimes [38]. It is unknown how the changes will impact non-natives in the SFE, though studies elsewhere show climate change is likely to benefit many invasives [3].

We investigated the relationship between temperature and salinity and *C. caspia* growth rates through a controlled laboratory experiment using field-collected individuals to better predict how the invasive population of *C. caspia* in the SFE may respond to changes in climate conditions. This approach is simple, yields conclusive results, and can be applied to any invasive species that is amenable to lab or greenhouse culture, such as plants and invertebrates with short generation times. Over a large range of temperatures, biologically we know the shape of the curve describing the relationship between growth rate and temperature/salinity should peak at the optimum and decrease at extreme conditions. We, however, were interested in investigating the relationship over the range of naturally occurring conditions and those predicted under a conservative climate change scenario (+2 C). The following hypotheses represent different possible relationships between growth rate and temperature/salinity over the current and predicted range:

Hypothesis 1: Growth rate is constant across the current and predicted range of temperature and salinity.

Hypothesis 2: Growth rate increases linearly with increasing temperature and/or salinity.

Hypothesis 3: Growth rate increases initially with increasing temperature and/or salinity and levels off at high temperatures, salinities, or both.

Hypothesis 4: Growth rate is unimodal, with maximal growth at intermediate temperature/salinity, and decreasing growth at the two extremes.

Through this work, we gain an understanding of the link between climate factors and the population growth rate of an important non-native species.

## Methods

### Ethics statement

All necessary permits were obtained for the described field collections (California Department of Fish and Game Scientific Collecting permit # SC-008862).

### Study area

*Cordylophora caspia* was likely introduced into the SFE system by ballast water exchange or ship hulls as early as the 1920s [11], [12]. It established populations in the brackish water habitats of the SFE, with the highest densities in Suisun Marsh. Suisun Marsh is a brackish water system covering approximately 34,000 ha in the upper San Francisco Estuary. One-third of the area is a system of tidally influenced sloughs, with margins of tules (*Schoenoplectus* spp.) and other brackish-water marsh plants, while the rest is a combination of diked seasonal pools and upland grasslands [30].

### Temperature×salinity experiment

On June 12, 2008, we collected *C. caspia* polyps from Suisun Marsh via settling plate arrays (100 cm2 sheet PVC plates, roughed with an orbital sander on both sides and hung below the water surface to allow *C. caspia* settlement), maintained them in 6.5 ppt salinity water in an aerated tank, and fed them with an overabundance of 48 hour old Self-Emulsifying Lipid Concentrate (SELCO) enriched *Artemia* nauplii. On July 27, 2008, we put 3 polyps each into 57 new petri dishes, containing 6.5 ppt water held at 20 C, by gently plucking them off the settling plates with forceps and placing them in the petri dish. We allowed polyps 3 days to settle in the petri dish, providing them with *Artemia* nauplii every other day. After settlement, we recorded the number of successfully settled polyps per petri dish. In six of the petri dishes, no polyps successfully settled, so these were not used. In four dishes, there were no feeding hydranths remaining, but there was live, intact stolon settled in the dish. We used these in the experiment and counted this as one settled polyp. The resulting sample size per temperature treatment was 18, 16, and 17 for 15, 20 and 25 C respectively, and 17, 18, 16 samples for 2, 6.5, and 11 ppt salinity treatments. We then randomly placed each of the 51 petri dishes containing polyps at the bottom of one of 36 5-gallon tanks holding 6.5 ppt water at 20 C, 1–2 petri dishes per 5-gallon tank. All 5-gallon tanks were randomly placed inside 1 of 6 large water baths to maintain the desired experimental temperature, each water bath holding 6 5-gallon tanks. There were two water baths per temperature treatment. Salinity treatments were achieved by adding Liquid Ocean to fresh water to obtain salinities of 2, 6.5 and 11 ppt. Each of the six 5-gallon tanks were randomly assigned to one of the salinity treatments, with two tanks per salinity treatment per water bath. Each tank was aerated with an aquarium aeration system. These treatment conditions were chosen to represent the range of conditions found in Suisun Marsh during the summer *C. caspia* growing season, with the upper bracket representing the temperature increase expected under a conservative climate change scenario [39].

On July 31, 2008 we began to adjust the salinities to reach the desired level. To avoid salinity shock, we altered the salinities by only 1 ppt every 4–12 hours. Once the polyps had a chance to acclimate to their new salinities, we began the temperature adjustment. We achieved the desired temperatures by slowly dropping or rising by 1 C every 1–3 hours over 24 hours. We fed the polyps to satiation every day on 48 hour old Selco enriched *Artemia* nauplii and executed 1/3 water changes of the tanks every other day to decrease fouling. We ran the experiment for 14 days and then, on August 13, 2008, we removed all the dishes from their experimental tanks and recorded the number of live hydranths (a feeding individual) in each dish using a dissecting microscope. All new hydranths observed in the experiment occurred through asexual propagation.

### Data analyses

Data were log-transformed and analyzed using a mixed model ANOVA to determine the effects of temperature and salinity on polyp colony growth. Due to the variation around the number of polyps that settled for the growth experiment (between 1–2 polyps successfully settled per petri dish) we used the proportional increase in polyps (final number of polyps/initial number of polyps) per dish as the dependent variable in the ANOVA analysis.

We used a model comparison approach to test our hypotheses about the affect of temperature and salinity on intrinsic growth rate, which describes the population's potential for instantaneous increase with unlimited resources. The shape of the relationship between the environmental condition of interest and growth rate is very important for predicting how an invasive species' population will persist or grow in the future. If the relationship is non-linear over the range of temperatures expected, then normal fluctuations in temperature may lead to non-intuitive effects on growth rates. The general phenomenon that the mean of a non-linear function of a variable is not the same as the function of the variable's mean, 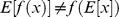, where *x* is a random variable and *f* is a non-linear function, is known as Jensen's inequality. This rule is important for evaluating the relationship between abiotic factors and biotic processes [40], [41]. In particular, an increase in an environmental condition, such as temperature, can lead to a larger than expected increase in growth rate if researchers fail to take Jensen's inequality into account.

To investigate the relationship between temperature/salinity and growth rate, we built a deterministic model corresponding to each hypothesis mentioned in the introduction. All the models used an exponential growth equation, 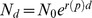, where N_d_ is the number of hydranths at time d, N_0_ is the initial number of hydranths, and d is the time in days. We represent the different hypotheses for the relationship between temperature and salinity and intrinsic growth rate in r(p). For hypothesis 1 (constant growth rate), r was a constant. For hypothesis 2 (linear increase), we modeled linear relationships between temperature and growth rate, between growth rate and salinity, between temperature, salinity, and growth rate, and the interaction between temperature and salinity. For hypothesis 3 (increasing growth rate but decreasing slope), we used the Michaelis-Menten function: 
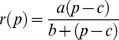
. In this parameterization, p is the condition tested (temperature or salinity) and a and b are estimated parameters. Since it is unlikely that growth rate goes to zero at exactly 0 C or 0 ppt, we also incorporated an additional parameter, c, to allow the growth rate to go to 0 at larger than 0 C or ppt. To represent hypothesis 4 (decrease in growth rate at the upper and lower end of temperature/salinity), we used a quadratic exponential (hump shaped) curve: 
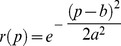
, again p is the condition tested and a and b are estimated parameters. This is a Gaussian curve without the normalizing term. Table 1 outlines the model parameterizations. We chose the Michaelis-Menten and the quadratic exponential curves as phenomenological representations for the pattern of interest. These models allowed us to distinguish between the different relationships that would arise from the different hypotheses. We used the estimated intrinsic growth rates (r) to estimate population doubling time at different temperatures and salinities: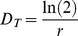.

**Table 1:** The results of the model comparison; the number before each hypothesized relationship corresponds with a specific hypothesis given in the introduction.

Hypothesis	Model	ΔAIC
3. Michaelis-Menten relationship with temperature	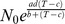	0.0
2. Linear relationship with temperature	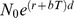	4.7
2. Linear relationship with temperature and salinity	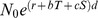	6.5
1. No impact of temperature or salinity	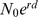	21.6
2. Linear relationship with salinity	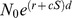	22.7
4. Quadratic exponential relationship with temperature	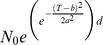	27.5
2. Linear relationship with temperature and a temperature/salinity interaction	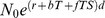	33.0
2. Linear relationship with temperature and salinity interaction	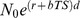	34.3

T = temperature, S = salinity, d = time in days, and r, a, b, c, and f are estimated parameters. In models for hypotheses 1, 2, and 3, r is the growth rate when temperature and salinity are zero. ΔAIC is the difference in AIC units between the model with the best AIC and the given model. Thus the best model has a ΔAIC of zero.

We fit these models to the data using maximum likelihood estimation assuming a negative binomial distribution of offspring because we started with small population sizes (1–2 polyps per dish) and thus expected demographic stochasticity to be important. Additionally, the negative binomial distribution allows for individual heterogeneity and the variance in the number of polyps produced at the end of the experiment was more than ten times its mean, indicating our data were over-dispersed. We compared the predictive ability of the fitted models using Akaike's Information Criterion (AIC).

## Results

We found a significant effect of temperature on growth of the polyp colonies (p = 0.0007), but no effect of salinity (p = 0.51) with the ANOVA analysis. The average growth rates at the increasing temperature levels were 0.26, 2.42, and 4.15 hydranths per polyp per day (standard error ±0.74, 0.47, 0.83 respectively). The maximum number of hydranths grown per polyp per day was 12.21 in the 25 C treatment.

**Figure 1:**
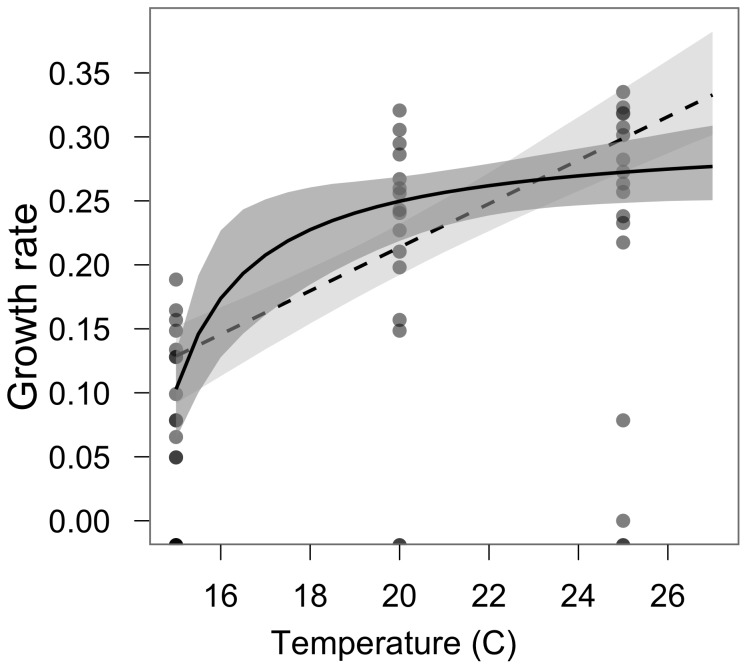
Modeled relationships between growth and temperature. Solid line represents the predicted Michaelis-Menten relationship between temperature and growth rate. Dashed line shows the predicted linear relationship between temperature and growth rate. Shaded areas demonstrate the 5^th^ and 95^th^ percent confidence interval for the predicted relationships. Circles show the observed data points. Increased darkness of circle shading corresponds with the number of data points at that location. Circles falling below 0.00 on the y-axis represent polyps that died (i.e., negative growth).

We found the Michaelis-Menten model (Hypothesis 3) with temperature best explained the variation in colony growth rates (4.7 AIC units better than the next best model). The resulting maximum likelihood estimates for the parameters in this model are: a = 0.30 (95% confidence interval: 0.22, 0.70), b = 1.21 (0.37, 18.77), c = 14.38 (11.48, 14.71). The model with a positive linear relationship between growth and temperature was the next best in modeling the growth rate. The parameter estimates with this model are a = −0.13 (95% confidence interval: −0.26, 0.02) and b = 0.02 (0.01, 0.02). Table 2 provides the intrinsic growth rates and doubling times for each temperature using the Michaelis-Menten and linear models. Figure 1 shows the top two models fitted to the data and Table 1 provides the AIC scores for all the models tested. Salinity had very poor predictive power so we present the non-linear results only incorporating temperature.

**Table 2:** Estimated intrinsic growth rate and doubling time for the modeled Michaelis-Menten and linear relationships between temperature and growth rate.

	Michaelis-Menten relationship		Linear relationship	
Temperature	Intrinsic growth rate (r)	Doubling time (days)	Intrinsic growth rate (r)	Doubling time (days)
**15**	0.10 (0.6, 0.13)	6.93 (5.26, 10.78)	0.13 (0.09, 0.15)	5.33 (4.55, 7.56)
**20**	0.25 (0.22, 0.27)	2.77 (2.58, 3.17)	0.21 (0.19, 0.23)	3.30 (2.99, 3.61)
**25**	0.27 (0.25, 0.30)	2.57 (2.31, 2.78)	0.30 (0.27, 0.34)	2.31 (2.05, 2.54)

5% and 95% Confidence intervals are given in parentheses.

## Discussion

Our results show that climate change may favor *C. caspia* in the SFE because it has the potential to rapidly propagate if temperatures increase. The current mean water temperature in the San Francisco Delta is 16 C and it is predicted to rise to 18–20 C by 2090 [37]. Climate change scenarios also predict increases in temperature variability [42]. The non-linearity we found in the relationship between growth rate and temperature signals that a relatively small increase in temperature experienced by *C. caspia* during its growing season could lead to a large increase in population growth during the growing season and in the length of the growing season. This could result in increased negative effects on the SFE system. The increase in growing season recently experienced by *C. caspia* in United Kingdom is thought to have lead to increased biofouling [22].

*C. caspia* is currently present in the brackish water habitats of the SFE system year round [43], [44]. The sharp downward drop in *C. caspia* growth rate near 16 C (Fig. 1) has the effect of strongly reducing the length of growing season and growth rate during the growing season under current temperature ranges. Our model shows *C. caspia* experiences positive growth rates at temperatures above 14 C and the peak growth occurs at temperatures above 19 C. Presently, the average water temperature in Suisun Marsh is above 14 C for 9 months of the year and under the modest climate change scenario (+2 C), it will be above 14 C for 10 months [45], resulting in an additional month when *C. caspia* has positive growth rates. Water temperatures in Suisun Marsh are also predicted to be above 20 C for many more days than currently occurs [39]. In Suisun Marsh, the average temperature is at the peak *C. caspia* growth temperature (above 19 C) from June through October [45]. Under the modest climate warming model (an increase of 2 C), this peak growth period would increase to May through November. The overall effect of climate change, therefore, will be to allow *C. caspia* to have a longer growing season, higher growth rates for a greater portion of the year, and its mean growth rate will increase disproportionately relative to the increase in temperature.

The growth rate during the growing season likely dominates the annual growth rate as *C. caspia* has the ability to form a diapause stage, called menonts, which allows tissue to remain dormant inside stems and stolons when conditions are unfavorable [46]. After favorable conditions resume, the menonts are able to regenerate more polyps and resume growth and reproduction. We know of no work that has investigated the survival rate of these menonts through the winter, though studies of methods for controlling *C. caspia* populations show the ability to form menonts makes eradication of *C. caspia* difficult [23], [24], [47].

We found, within the ranges tested, salinity did not affect colony growth rate. Suisun Marsh is predicted to experience increased variability in salinity with climate change [36]. While this is likely to have a negative impact on some invasives, such as non-native clams, and may favor important fish species [36], our study shows that this is unlikely to have a negative impact on *C. caspia* populations. Other studies have also found *C. caspia* has a wide range of salinity and temperature tolerance limits, but the actual limits vary with the populations studied [24], [43], [46], [48], [49]. It has also been shown that *C. caspia* has the ability to structurally remodel itself, from the cellular level up to colony formation, in response to changes in salinity in order to maintain metabolic function (reviewed in [50]). Our results add to this body of work by showing that while *C. caspia* is likely to benefit from a warmer climate, it is unlikely to be harmed by the predicted changes in salinity, making it quite likely that it will thrive under the new conditions.

There are other factors, both abiotic and biotic, that we have not investigated that may limit *C. caspia* abundance. This includes the availability of settling habitat. However, studies have shown that *C. caspia* is very adept at taking advantage of available settling habitat, including settling on live cyprinid fish [24], [43], [46]. During the height of the *C. caspia* summer growing season, Wintzer et al. [43] estimated maximum recruitment to newly available settling habitat at 22,998 hydranths per day per m^2^ in some areas of Suisun Marsh. However, while *C. caspia* are very successful colonizers, they may be easily outcompeted for space after initial settlement [43], [51]. In the SFE, *C. caspia* has to compete with other non-native fouling species, such as the Australian tubeworm (*Ficopomatus enigmaticus)*, the common sea grape (*Molgula manhattensis)*, the bay barnacle (*Balanus improvises)*, and an unidentified bryozoan [43].

Food resources and predators may also limit population growth. *C. caspia* may be limited by zooplankton availability, as there has been a decreasing trend in biomass of some native zooplankton species, but this may be offset by an increasing trend in non-native zooplankton biomass [52]. The non-native shimofuri (*Tridentiger bifasciatus*) and shokihaze (*T. barbatus*) gobies are known to be *C. caspia* predators in the SFE [44], [53], [54]. Additionally, the aeolid nudibranch, *Tenellia adspersa*, which is also native to the Ponto-Caspian region, preys upon *C. caspia* and was found in large quantities on our settling plates in Suisun Marsh [55], [56]. Our study shows warmer temperatures will likely result in increased growth rates of C. caspia, which may in turn promote growth rates of their non-native predators through increased prey availability. It will be important to investigate how these interactions may be altered by climate change and evaluate which non-natives may be favored or limited under future conditions.

Our method of experimentally studying the relationship between environmental factors and population growth rate provided valuable insights into the ecology of *C. caspia* and how it might be affected by climate change. Through our study, it is clear that the environmental factors predicted to change under climate change are likely to promote *C. caspia* population growth. The understanding that can be gained from experimental studies of demography such as this should not be overlooked.

The experimental approach we employ has the benefit of quantifying a piece of the puzzle that will determine non-native species' abundances and distributions under climate change: the relationship between population growth rate and climate variables [10], [57]. Laboratory experiments such as this one should be employed more often to investigate the direct relationship between population growth rates and climate variables predicted to change under climate change. The results can then be incorporated in hybrid niche models, which combine a mechanistic understanding with phenomenological models, in order to gain a more complete picture of how non-native species' populations will be altered by climate change [7], [10], [58]. An informative extension of this study will be to incorporate the mechanistic understanding gained with an environmental niche model to further predict how the invasive population of *C. caspia* may change in both extent and abundance.

### Conclusions

Our experiments found that *C. caspia* growth rates increased non-linearly with temperature and were not influenced by salinity. This non-linear relationship predicts that a small increase in temperature can result in a large increase in growth rate by reducing the proportion of time *C. caspia* growth is limited by temperature. If *C. caspia* populations in the SFE increase in abundance with climate change, as suggested by our results, they may negatively influence the zooplankton community or become a biofouling nuisance, as they have in other systems. This impact may be detrimental to native planktivorous fish populations, which also depend on zooplankton as a food source. This study highlights the valuable insights that can be gained through direct experimental investigations of environmental conditions and population growth rates.
